# Screening for autism identifies behavioral disorders in children functional defecation disorders

**DOI:** 10.1007/s00431-016-2775-x

**Published:** 2016-09-13

**Authors:** Sophie Kuizenga-Wessel, Carlo Di Lorenzo, Lisa M. Nicholson, Eric M. Butter, Karen L. Ratliff-Schaub, Marc A. Benninga, Kent C. Williams

**Affiliations:** 1 Department of Pediatric Gastroenterology, Emma Children’s Hospital AMC, H7-250, PO Box 22700, Amsterdam, 1100 DD The Netherlands; 2 Department of Pediatric Gastroenterology, Nationwide Children’s Hospital Columbus, Columbus, OH USA; 3 Department of Health Research and Policy, School of Public Health, University of Illinois at Chicago, Chicago, IL USA; 4 Department of Behavioral Health, Child Development Center, Nationwide Children’s Hospital, Columbus, OH USA

**Keywords:** Constipation, Autism spectrum disorders, Pediatrics

## Abstract

This study prospectively assessed whether positive screening surveys for autism spectrum disorders (ASDs) in children with functional defecation disorders (FDDs) accurately identify ASD. Parents of children (4–12 years) who met Rome III criteria for functional constipation (FC), FC with fecal incontinence (FI) and functional nonretentive FI (FNRFI) completed two ASD screening surveys. Children with positive screens were referred for psychological evaluation, and a year later, follow-up surveys were conducted. Of the 97 study participants, 30.9 % were diagnosed with FC, 62.9 % with FC with FI, and 6.2 % with FNRFI. ASD surveys were positive for 27 children (27.8 %). New DSM diagnoses were made in 10 out of the 15 children that completed further evaluation. Two (2.1 %) met criteria for ASD, and 12 (12.4 %) met criteria for other behavioral disorders. Average SRS and SCQ-L scores were higher in subjects with FC with FI as compared to FC alone and in those who reported no improvement versus those who reported improvement 1 year later.

*Conclusion*: While positive ASD screening surveys did not correctly identify ASD in the majority, it did help to identify other unrecognized behavioral disorders in children with FDD. High screening scores were more common in children with FC with FI and in children with poorer responses to current medical treatments.

**Table:** 

**What is Known:** *•A prior study found that 29 % of children with FDD scored positive on ASD screening questionnaires.* *•Whether positive screens correctly identify ASD in children with FDD is unknown.*
**What is New:** *•This study shows that positive ASD screens do not correctly identify ASD in children with FDD. However, the use of ASD screening questionnaires can identify previously unrecognized and untreated behavioral/developmental disorders in children with FDD.* *•High screening scores are more common in children with FC with FI and in children with poorer responses to current medical treatments.*

## Introduction

Functional defecation disorders (FDDs) are common in children with an estimated prevalence of functional constipation (FC) in children worldwide ranging from 0.7 to 29.6 % [18]. FDD include FC, FC with fecal incontinence (FC + FI), and functional nonretentive fecal incontinence (FNRFI) [23]. The main characteristics of FC are infrequent, and hard and painful defecations often accompanied by the involuntary loss of feces in the underwear. FDDs seem to be particularly common in children with behavioral and developmental disorders, such as attention deficit hyperactivity disorder (ADHD) and autism spectrum disorders (ASDs) [7, 14, 16]. In an effort to better understand the association between FDDs and ASD, Peeters et al. used two validated ASD screening surveys (Social Responsiveness Scale (SRS) and the Social Communication Questionnaire–Lifetime (SCQ-L)) to prospectively screen children with functional defecation disorders (FDDs) [20]. They found that 29 % of children with FDDs had ASD symptoms. It is uncertain if these children indeed had ASD, since study patients were not referred for behavioral and psychological assessment.

ASD is a neurodevelopmental disorder, defined by persistent deficits in social communication and interaction, as well as by restricted and repetitive patterns of behaviors and activities according to the Diagnostic and Statistical Manual of mental disorders–Fifth Edition (DSM-5) [1]. The diagnosis of ASD cannot be based on high rates of ASD symptoms in screening surveys; a full child psychiatric assessment is needed. Whether a positive ASD screen in children with FDD accurately identifies children with ASD is unknown.

Our primary objective was therefore to prospectively assess whether positive ASD screening surveys (SRS and SCQ-L) in children with FDDs accurately identify ASD.

While the SRS and SCQ-L [8, 20, 24] are often used to screen for ASD in children, the results from these screens may be influenced by the presence of internalizing and externalizing behaviors not associated with an ASD [13]. Interestingly, studies assessing psychosocial factors associated with FDDs in children have found an increase of both internalizing and externalizing behaviors in these children [22, 28]. The presence of these behaviors, in particular externalizing behaviors, was associated with differences in response to treatments for FDDs [28].

Our secondary aims were to evaluate if other DSM-5 disorders are present in children with positive ASD screenings surveys, but not having ASD, as well as whether results of ASD screens varied among various FDD diagnoses and between responders and nonresponders 1 year after recruitment.

## Methods

A prospective cohort study was carried out between August 2012 and February 2013. Children (4–12 years), who met criteria for a diagnosis of FC, FC with FI, or FNRFI based upon Rome III criteria [23], were recruited from the tertiary outpatient clinic of pediatric gastroenterology of the Nationwide Children’s Hospital (Columbus, OH). Patients were included regardless of previous treatments for constipation or previously diagnosed behavioral and developmental health problems. Patients were excluded if they had a medical disease that could have contributed to the development of constipation, such as inflammatory bowel disease, celiac disease, congenital anorectal malformation, history of colonic surgery, cerebral palsy, and spina bifida. After informed consent was obtained, parents of caregivers were asked to complete the two ASD screening surveys: SRS and SCQ-L. This study was approved by the Nationwide Children’s Hospital Institutional Review Board.

### Social Responsiveness Scale

The SRS is a 65-item scale that requires parents to rate the child’s behaviors in the previous 6 months in 4- to 18-year olds. The questionnaire focuses on behaviors grouped into categories of social cognition, social motivation, social communication, social awareness, and autistic mannerisms [9]. Each question is rated on a four-point Likert scale ranging from 0 (never true) to 3 (almost always true), generating a total score in the range from 0 to 195. Total raw scores can be transformed into gender-normed T-scores with higher scores indicating a greater severity of behavioral symptoms. A total score ≥51 is suggestive for the presence of ASD [8].

### Social Communication Questionnaire–Lifetime

The SCQ-L consists of 40 yes/no questions to evaluate behaviors associated with social interaction, communication, abnormal language, and stereotypical behaviors. Patients should be over 4 years, with a minimum mental age of 2 years. Total scores range from 0 to 40. A total score of ≥15 is suggestive for the presence of ASD [24].

### Behavioral evaluations

Children who met the preestablished cutoff scores for one or both questionnaires were referred to a clinical psychologist or clinical social worker from the Child Developmental Center at Nationwide Children’s Hospital for a comprehensive behavioral health diagnostic assessment. Patients were not only evaluated for whether they met criteria for ASD but also for other DSM-5 behavioral disorders. Patients were only included when all questionnaires were completed.

### Clinical course

A year after enrollment, follow-up surveys were mailed to the home of all participants with a self-addressed stamped envelope for returning the survey to clinical investigators. The survey contained the questions about bowel movements from the Rome III Diagnostic Questionnaire for the Pediatric Functional GI Disorders that were utilized for making the initial FDD diagnoses. The survey also asked if the children were taking medications or supplements for treatment of constipation. Based upon the responses, children were categorized as those whose bowel patterns still met criteria for an FDD and those whose bowel patterns no longer met criteria for an FDD.

### Outcomes

The primary outcome was the number of children with ASD diagnoses, confirmed by psychological evaluation, within the group of positive SRS and SCQ-L scores. Secondary outcome was the number of children with positive SRS and SCQ-L scores with other DSM disorders, as well as whether differences exist in scores of ASD screening surveys among different FDD diagnoses and between the outcome groups of responders and nonresponders. We hypothesized that if the severity of abnormal behavior is associated with fecal incontinence, scores for SRS and SCQ-L would be higher for those with FC+FI and/or FNRFI than those with FC. In addition, if abnormal behaviors are associated with poorer prognosis, then one would expect SRS and SCQ-L scores to be higher for those who still met criteria for an FDD (nonresponders) as compared to those who were improved (responders) a year after enrollment in the study and no longer met criteria for an FDD.

### Statistical analyses

For statistical analyses, Stata S.E. version 13.1 was used. Total raw scores on the SRS and total raw scores on the SCQ-L were analyzed as continuous variables. Independent sample *t* tests were used to examine differences in means in continuous variables across groups of children who improved compared to those that did not. Chi-squared analyses with Fisher’s exact tests were performed to compare proportions of categorical variables. The significance level was set at *p* < 0.05.

## Results

### Characteristics of the study sample

A total of 127 consecutive patients referred for evaluation and treatment of FDDs were asked to participate. Thirty patients were excluded, because they did not meet inclusion criteria (*n* = 16) or parents were not interested to participate (*n* = 14). Parents of 97 patients completed both ASD screening questionnaires (Table 1). Among the 97 study participants, 63 % (*n* = 61), 31 % (*n* = 30), and 6 % (*n* = 6) fulfilled the Rome III criteria for FC, FC+FI, and FNRFI, respectively.

**Table 1:** Patient characteristics

	Total	Negative screens	Positive SRS	Positive SCQ-L
Number of participants	97	70	27	8
Gender, *n* (%)				
Male	53 (55)	39 (56)	14 (52)	6 (75)
Female	44 (45)	31 (44)	13 (48)	2 (25)
Mean age in years (SD)	7.8 (±3.0)	8 (±3.2)	7.4 (±2.3)	7.3 (±2.3)
Diagnosis FDD, *n* (%)				
FC	30 (31)	25 (36)	5 (9)	0 (0)
FC with FI	61 (63)	40 (57)	21 (78)	8* (100)
FNRFI	6 (6)	5 (7)	1 (4)	0 (0)
Diagnoses of ASD				
Prior diagnosis of ASD	1	0	1	1
ASD suspected	1	0	1	1
Previously diagnosed behavior disorder (parent reported)	14	4	10^#^	2
Taking medication for behavioral issues	8	3	5**	2

As compared to participants with negative screens, higher percentage of positive SCQ-L participants were diagnosed with FC with FI (*p value = 0.024) and higher percentage of positive SRS participants had behavioral disorders (#*p* value = 0.000) and took medications for behavioral issues (**p value = 0.036)

*ASD* autism spectrum disorder, *FC* functional constipation, *FC with FI* functional constipation with fecal incontinence, *FNRFI* functional nonretentive fecal incontinence, *SRS* Social Responsiveness Scale, *SCQ-L* Social Communication Questionnaire–Lifetime

Initial medical histories revealed that 16 of the 97 (16.5 %) participants presented with a previously diagnosed or suspected developmental and/or behavioral disorder by medical providers from other institutions. One patient presented with an ASD diagnosis of pervasive developmental disorder—not otherwise specified (PDD-NOS). An ASD diagnosis was suspected in another participant. One or more behavioral disorders were reported in another 14 patients, including ADHD (*n* = 9), oppositional defiant disorder (ODD) (*n* = 5), anxiety disorders (*n* = 4), obsessive compulsive disorder (OCD) (*n* = 1), and disruptive behavior disorder–NOS (*n* = 1) (see “previously diagnosed behavior disorders” in Table 2). Of the 14 patients with a previously diagnosed disorder not fitting under ASD, 8 were taking medications for their behavioral issues.

**Table 2:** Results of behavioral evaluation

Patient	Gender	Age	Rome III diagnosis	SRS score	SCQ-L score	Previously diagnosed behavior disorder	Behavior diagnosis based on study evaluation
1	F	5	FC with FI	112	*29*	PDD-NOS	PDD-NOS
2	M	10	FC with FI	96	*17*	None	Asperger’s^a^, ADHD^a^, DBD-NOS^a^
3	F	4	FC with FI	92	*16*	ASD (suspected)	22q11.2 deletion^a^, learning disorder^a^, DBD-NOS^a^
4	M	8	FC with FI	124	*18*	ADHD, OCD, GAD, ODD	ADHD, ODD
5	M	6	FC with FI	141	*22*	ADHD, ODD, PTSD	ADHD, ODD, PTSD
6	M	9	FC with FI	127	*21*	None	ADHD^a^, DBD-NOS^a^
7	M	6	FC with FI	102	*16*	None	Learning disorder^a^
8	M	10	FC with FI	104	*19*	None	No diagnosis
9	M	10	FC with FI	71	3	ADHD, ODD	ADHD, ODD
10	F	5	FC with FI	60	5	GAD	GAD, separation anxiety disorder^a^
11	F	5	FNRFI	84	9	None	DBD-NOS^a^
12	F	5	FC with FI	59	4	None	ADHD, ODD^a^
13	F	8	FC with FI	54	6	None	Anxiety disorder NOS^a^
14	F	7	FC with FI	83	8	None	Adjustment disorder^a^, ADHD^a^
15	F	8	FC with FI	91	6	None	ADHD^a^, anxiety disorder^a^

Values in italics are positive SCQ-L scores

*ASD* autism spectrum disorder, *FC* functional constipation, *FC with FI* functional constipation with fecal incontinence, *FNRFI* functional nonretentive fecal incontinence, *PDD-NOS* pervasive developmental disorder–NOS, *ADHD* attention deficit hyperactivity disorder, *OCD* obsessive compulsive disorder, *GAD* generalized anxiety disorder, *ODD* oppositional defiant disorder, *PTSD* posttraumatic stress disorder, *DBD-NOS* disruptive behavioral disorder–NOS

^a^New diagnosis

The majority of patients was referred (*n* = 83) by their primary care physicians, five by the emergency department, and the other nine by other specialists within the hospital; none was referred by another pediatric gastroenterologist.

### Autism spectrum disorder screening questionnaires

Among the 97 study participants, 27 (27.8 %) scored above the cutoff value for one of the two ASD screening questionnaires (Table 1). All 27 had SRS scores ≥51, and 8 of the 27 had a SCQ-L ≥15. The most common FDD among the 27 with a positive SRS was FC+FI (*n* = 21), followed by FC (*n* = 5) and FNFRI (*n* = 1). All eight participants with a positive SCQ-L had FC+FI. The distribution of FDD diagnosis did not significantly differ between patients with positive and negative screening questionnaires (*p* = 0.168). Compared to those who scored <51, children with SRS scores ≥51 had a higher percentage of previously diagnosed behavioral disorders and of medications use for behavioral issues (Table 1). As shown in Table 1, four patients with previously known behavioral disorders scored negative on ASD screening questionnaires.

All 27 children with positive ASD questionnaires were referred for further evaluation. Parents of 12 patients were not interested in a referral and were therefore lost to follow-up. Half of these 12 patients were previously diagnosed with behavior disorders: 3 patients had ADHD and used medication, 1 was diagnosed with disruptive behavior disorder–NOS, and 2 had an anxiety disorders, of which 1 was seeing a therapist.

### Results of behavior evaluations

Subsequently, 15 participants (55.6 %) were seen by a psychologist or clinical social worker to undergo evaluations for behavioral and developmental disorders (Table 2).

#### Previously known diagnoses

The histories obtained from the parents/caretakers for these 15 children revealed that providers from other institutions had previously diagnosed 5 with a developmental or behavioral disorder (1 with ASD and 4 with behavioral disorders). A primary care physician had recently referred one child for evaluation of a suspected ASD. The remaining nine had never been diagnosed with or previously suspected to have a behavioral or developmental disorder (Table 2).

#### New or confirmed diagnoses by developmental evaluation

Developmental evaluations found that 14 of the 15 patients met criteria for developmental, behavioral, and/or learning disorders. New diagnoses were made in ten patients. However, developmental evaluations demonstrated that only 2 of the 15 children met criteria for an ASD. One of the two was the participant who was previously diagnosed with ASD at an outside institution. The other one had never been suspected of having an ASD and was diagnosed with Asperger’s disorder. As for the child who was previously suspected to have an ASD, the evaluation resulted in a diagnosis of disruptive behavioral disorder–NOS and a learning disorder. In addition, genetic testing discovered a 22q11 deletion.

Next to the two ASD diagnoses, positive ASD screens led to diagnosis of various types of other disorders. Disorders of externalizing behaviors were identified in ten patients, including ADHD (*n* = 8), ODD (*n* = 4), and disruptive behaviors–NOS (*n* = 4). Anxiety disorders were identified in three patients, including generalized anxiety disorder (*n* = 1), anxiety disorder (*n* = 2), and separation anxiety (*n* = 1). Other diagnosis made were learning disorders (*n* = 2), adjustment disorder (*n* = 1), and posttraumatic stress disorder (*n* = 1)(Table 2).

### Scores according to the functional defecation disorder

**Fig. 1:**
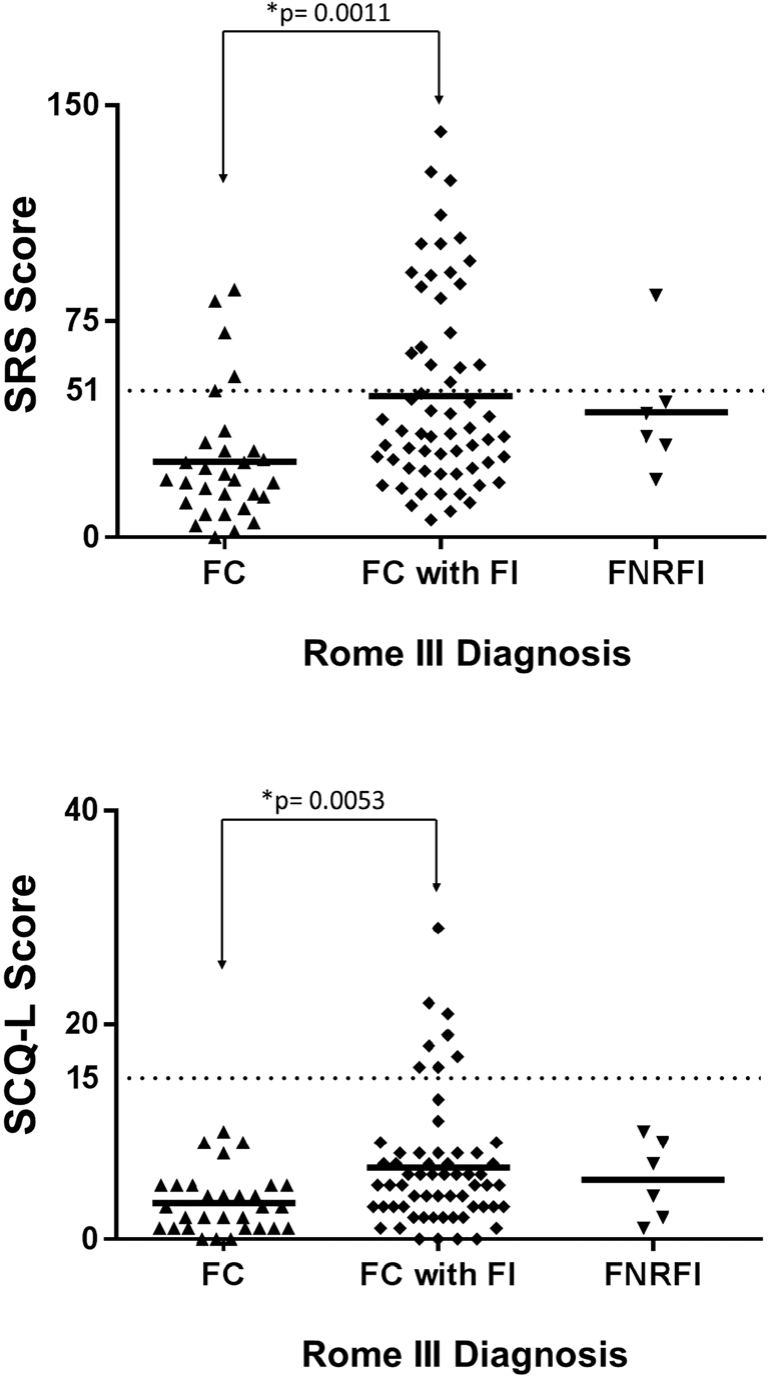
Scores ASD screening questionnaires compared to FDD diagnosis

The average scores for SRS (49.2 ± 33.7) and SCQ-L (6.7 ± 3.4) of children diagnosed with FC+FI were statistically higher than the average scores for SRS (26.3 ± 22.3, *p* = 0.0011) and SCQ-L (3.4 ± 2.8, *p* = 0.0053) of children diagnosed with FC (Fig. 1). The average scores of children with FNRFI did not differ significantly from the average scores of either FC+FI or FC groups of children. Of note, all eight participants who were positive for both SRS and SCQ were diagnosed with FC+FI.

### Scores according to clinical course—1-year follow-up

Among the initial 97 participants, parents/caregivers for 52 (53.6 %) responded to the survey sent them 1 year after enrollment. Parental responses to questions on bowel patterns indicated that bowel patterns for 26 of the 52 (50 %) no longer met criteria for an FDD while the bowel patterns for the other 26 (50 %) continued to meet criteria for an FDD. Of the 26 whose bowel patterns no longer met criteria for an FDD, 9 reported that they were taking medications for constipation while 17 reported not being on any laxatives/supplements for constipation. Of the 26 who still met criteria for an FDD, 17 reported that they were taking medications for constipation while 9 reported not being on any laxatives/supplements for constipation.

Comparison of scores between groups revealed that at the time of enrollment the average scores for the SRS (46.7 ± 30.8 vs. 31.6.2 ± 27.6, *p* = 0.034, one-tailed test) and the SCQ-L (6.3 ± 4.7 vs. 4.2 ± 4.2, *p* = 0.049, one-tailed test) were significantly higher in those who still met criteria for an FDD after 1 year as compared to those who no longer met criteria for an FDD after 1 year.

## Discussion

The 27.8 % positive ASD screens in the current study population of children with FDDs closely match previous findings [20]. However, formal psychological evaluations found that positive SRS and SCQ-L screens were not able to correctly identify an ASD diagnosis in our study population, but did identify children with previously undiagnosed behavioral disorders. Findings from the current study indicate that children with FDDs display behaviors that are common to ASD and other behavioral disorders. The use of ASD screening surveys resulted in ten new DSM diagnosis that were unknown or unrecognized by parents and previous healthcare professionals. Furthermore, our findings indicate that children with FC+FI have a higher severity of abnormal behaviors that result in higher survey scores than children with other FDDs. In addition, comparison of scores indicate a higher prevalence of abnormal behaviors in children who do not improve with standard medical interventions as compared to those that do improve.

Findings from the current study, along with previous reports, provide strong evidence that many children with FC+FI share a common behavioral phenotype that results in positive ASD screens. Prior studies have found that the presence of increased internalizing and externalizing behaviors are associated with higher SRS scores [13]. Attention deficit and disruptive behavior disorders, which were the most common group of disorders found in the current study, are disorders of externalizing behaviors. Externalizing behaviors have also been associated with fecal incontinence in children with ASD [21] and in the general pediatric population [15, 28]. Many of the abnormal behaviors for which the SRS and SCQ-L screen commonly occur not only in children with ASD but also in children with other behavior disorders. Towbin et al. [25] demonstrated that a substantial proportion of children with mood and anxiety disorders scored in the ASD-likely range of the SRS. Similarly, children with ADHD may find some aspects of social interaction and communication more difficult than healthy children [17]. In addition, when ASD is compared to disruptive behavior disorders, the sensitivity and specificity of the SRS is considerably lower [17].

While use of SRS and SCQ-L may not accurately identify ASD in children with FDDs, the current study indicates, in accordance with previous studies [20, 28], that behavior problems are common in children with FDDs. Even though this study could overestimate the prevalence of behavior disorders in children with FDDs, due to the fact that parents concerned for the presence of behavior problems in their children are more likely to participate in this study, we believe that behavioral screening should be incorporated into the diagnostic workup of children with FDDs. Since screening could result in early diagnosis and treatment of previously unknown behavior disorders and may have therapeutic consequences. Based on our results, we cannot advise which screening surveys should be used. We can only speculate that it might be better to use more general surveys that screen for both internalizing and externalizing disorders, rather than ASD-specific screens. However, future research is necessary to answer this question.

In addition to being associated with a particular type of FDD, the results from the 1-year follow-up survey indicate that behaviors that the ASD screens measure are associated with poorer outcomes. This observation confirms previous reports that indicate that increased behavioral and social problems are associated with the need for longer treatment and poorer outcome in children with FDDs [16, 28, 29]. Identifying previously unrecognized behavioral disorders would provide opportunity to address such issues that may be contributing to the poorer response to routine medical interventions in this patient population. However, it is possible that the poorer outcomes are related to noncompliance with FDD treatment regimens.

While prior studies have shown mixed results, the overall use of behavioral interventions in addition to common laxative therapies appear to produce slightly higher rates of improvement in the treatment of FDD, particularly when treating FC+FI [2, 4, 6, 19, 27, 29]. However, even in studies where combined behavioral and laxative therapy produced the best outcomes over other treatment regiments, the rates of cure at 12-month follow-up are at best 51 % [4, 6, 19]. In this study, we also reported that 50 % no longer met the Rome III criteria for FDDs at follow-up. Such disappointing cure rates suggest the need to better understand the clinical characteristics of children at risk of not responding to standard behavioral and medical therapies. The use of behavioral screens such as those in this study may not only help clarify specific behavioral phenotypes associated with FDDs, but may also lead to the development of specific behavioral and/or cognitive therapies needed to improve current outcome rates for FDDs. The relatively ineffectiveness of psychological treatment for FDDs could also be explained by the focus of behavioral therapy on fear of defecation. Based on the assumption that constipation often has to do with fear of defecation and consequently to withholding of stools, behavior therapy is mainly focused on these problems. Findings from current study along with previously published reports showed that children with FDDs also have externalizing behavior problems. Treatment results might improve if behavioral treatments are focused on both internalizing and externalizing behavior problems.

To date, it has remained uncertain whether behavioral problems are primary or secondary to FDDs. It can be hypothesized that preexisting behavior problems may lead to a complicated toilet training, which is known as a critical phase in the development of FDDs [5]. On the other hand, the presence of FDDs might give rise to a considerable level of stress and embarrassment for the child and family. Fecal incontinence triggers parental stress because of the dishonesty of the child about fecal accidents and the burden of cleaning clothes. In addition, the majority of parents assume that fecal incontinence is caused by the child’s laziness, carelessness, and stubbornness [3, 10, 11, 26]. This influences the parent-child interaction negatively and could therefore be related with the onset or maintenance of FDDs in children.

This study has several strengths. Diagnoses of FDDs in children were made according to the internationally accepted Rome III criteria [23]. Additionally, the diagnosis of ASD and other DSM diagnoses were made after behavioral and psychological assessment by experienced behavioral health clinicians. Nevertheless, there are limitations that should be taken into account when interpreting our results and extrapolating them to other patient cohorts. First, there could have been a selection bias toward more severe cases of FDDs because the study patients were recruited in a tertiary care center. This could have resulted in a higher percentage of behavioral health problems as children presenting at a tertiary care center might be more difficult to treat. Care should therefore be taken before generalizing these results to children who have FDDs and are treated in a general pediatric practice. However, most of prior healthcare provided was not from tertiary care centers as the majority (86 %) was referred and presenting from general pediatric practice. Another limitation was the relatively small sample size and the low response rate in the 1-year follow-up. Finally, the use of parents as informants to fill out the ASD screens and Rome III questions may be a confounder of this study, since parental report may also be biased by the parents’ own psychological or health status. However, the ASD screens used in this study have been previously validated and parent report of GI problems has been highly correlated with GI diagnoses made by physicians [8, 12, 24].

## Conclusion

Our findings draw attention to the fact that behavioral health problems are often unrecognized in children with FDD. While positive ASD screening surveys did not correctly identify ASD confirmed by psychological evaluation in the majority of children, it did help to identify other unrecognized behavior disorders. Presence of the behaviors uncovered by the ASD screens appears to be more common in children with FC+FI and in children with poorer responses to current medical treatments. Future studies are needed to determine whether early identification and therapy for externalizing behaviors associated with ASD and other behavioral disorders could help improve treatment outcomes for children with FDDs.

## Abbreviations

ADHD: Attention deficit hyperactivity disorder
ASD: Autism spectrum disorder
FC: Functional constipation
FDD: Functional defecation disorders
FI: Fecal incontinence
FNRFI: Functional nonretentive fecal incontinence
SRS: Social Responsiveness Scale
SCQ-L: Social Communication Questionnaire–Lifetime
